# Transcriptome Profile of Next-Generation Sequencing Data Relate to Proliferation Aberration of Nasopharyngeal Carcinoma Patients in Indonesia

**DOI:** 10.31557/APJCP.2020.21.9.2585

**Published:** 2020-09

**Authors:** Dicka Wahyu Setiasari, Gisti Rahmawati, Digdo Sudigyo, Risky Hiskia Poluan, Salsabila Lutfi Sesotyosari, Tirta Wardana, Sagung Rai Indrasari, Cita Herawati, Didik Setyo Heriyanto, Indwiani Astuti, Afiahayati, Sofia Mubarika Haryana

**Affiliations:** 1 *Study Program of Biotechnology, Graduate School, Universitas Gadjah Mada, Yogyakarta, Indonesia. *; 2 *Bioinformatics and Data Science Research Center, Bina Nusantara University, Jakarta, Indonesia. *; 3 *Faculty of Medicine, Public Health and Nursing, Universitas Gadjah Mada, Yogyakarta, Indonesia. *; 4 *Head and Neck Departement, Dharmais Cancer Hospital, Jakarta 11420, Indonesia. *; 5 *Department of Computer Science and Electronics, Faculty of Mathematics and Natural Sciences, Universitas Gadjah Mada, Yogyakarta, Indonesia. *

**Keywords:** Nasopharyngeal carcinoma, next-generation sequencing, transcriptome analysis, proliferation

## Abstract

**Objective:**

Nasopharyngeal carcinoma (NPC) is the most common cancer arising from epithelial cells of the nasopharynx in Indonesia. This study aims to determine the differential level of gene expression in NPC patients when compared with normal individuals. Transcriptome profiling analysis was performed using RNA-Seq technology to determine the differential gene expression relate to proliferation aberration that occurs in NPC patients compared with normal individuals. So we get the transcriptomic profile of Indonesia NPC patients.

**Methods:**

In this study, we used 9 samples, 7 NPC samples and 2 normal samples as control. Fresh tissue of tumor samples was collected from biopsy, and normal samples were collected brushing technique. The total RNA was isolated from fresh tissue samples and brushing samples using the Rneasy^®^ RNA Extraction Mini Kit. The cDNA library was generated using TruSeq^® ^RNA Library Preparation Kit V2, and its concentration was determined using qPCR. The library was sequenced using the Next-Generation Sequencing (NGS) Illumina Next Seq 550 platform. The raw sequence data quality was analyzed using FastQC and interpreted using HISAT2, HTSeq, edgeR, and PANTHER.

**Results:**

From the analysis, 25493 gene transcripts were expressed, with 1956 genes were significantly upregulated, 90 genes were significantly downregulated in NPC samples, and 23897 genes didn’t change the expression level significantly (P<0.05), 10 of which genes were associated with cell proliferation. These genes are involved in the regulation of cancer cell proliferation through several signaling pathways, which are the apoptosis signaling pathway, IGF signaling pathway, Notch signaling pathway, and P13K signaling pathway.

**Conclusion:**

There were significant differences in gene expression levels between NPC patients and normal individuals. Each gene that has changed the expression level plays a role in regulating various pathways that lead to cell proliferation aberration in NPC cases.

## Introduction

Nasopharyngeal carcinoma (NPC) is a type of cancer originating from squamous epithelium cells from the surface of the lateral nasopharyngeal wall. This cancer has unique epidemiology, with incidents varying according to race and geographical differences. NPC cases are mostly found in China and Southeast Asia with highly prevalent malignant diseases and are the leading cause of death cases in several regions in China and Southeast Asia. NPC cases rarely found in Europe, but more often found in certain ethnic groups, like Chinese who live in Europe (Brennan, 2006). Indonesia is one of the countries with a high prevalence of NPC patients and is the most frequent case after breast cancer, cervical cancer, and lung cancer. The prevalence rate of NPC cases in Indonesia reaches 6.2/100,000 or about 12,000 new cases per year (Adham et al., 2012). 

Risk factors for NPC are influenced by gender, genetic susceptibility, age, race, environment, habits, and Epstein-Barr Virus (EBV) infection. In men, the incidences are about 9,4/100,000 per year, while women are 3.8/100,000 per year (Sudiono and Irma, 2013). According to Simon (2011), on genetic susceptibility factors, each race has a specific Human Leukocyte Antigen (HLA) gene pattern. Certain HLA alleles in a population are associated with an increased risk of developing NPC. The HLA allele pattern has a strong association with the parallel genetic predisposition of NPC. The incidence of this cancer often attacks people in the productive age between 40-60 years old, and rarely attacks the young people and children (Downing and Wolden, 2009). However, Rahma and Muhtarum (2015) mention that in the last decade, there was an increased incidence of NPC at the age of 10-20 years old and began to attack many children. On environmental factors that play an essential role in triggering the activation of EBV, some carcinogenic compounds such as nitrosamine, benzopyrene, bensoanthracene, and several other types of hydrocarbons are thought to act as mediators in activating EBV. Besides, the compounds contained in cigarette smoke vehicle and industrial exhaust gases and other pollutants will increase the possibility of NPC (Adham et al., 2012).

The high mortality rate of NPC patients is due to the late diagnosis. NPC patients usually diagnosed when cancer has developed at an advanced stage, resulting in delayed treatment and causing a high risk of death. Therefore we need an early detection method that is fast and effective to reduce mortality. Through a molecular approach, the cellular condition of a specific tissue can be known, so the transcriptomic profile analysis is an appropriate method to know the gene expression pattern to determine the cellular status. In this study, the transcriptomic profile analysis was performed using RNA Sequencing (RNA-Seq) technology, that aims to determine the differential level of gene expression in NPC patients when compared with normal individuals. The genes that significantly changed their expression level in the case of NPC describe a signature of Indonesian NPC patients. So later, it can be used as a biomarker candidate for early and effective detection methods and as a therapeutic target for treatment based on gene therapy.

## Materials and Methods

We used 9 samples in this study that obtained consecutively, consisting of 7 NPC samples and 2 control samples. NPC samples were collected from the biopsy tissue of NPC patients. The biopsy tissues were taken by an ENT specialist at the Dharmais Cancer Hospital, Jakarta Indonesia. Control samples were collected from brushing on the nasopharyngeal area of normal individuals. The brushing samples were collected by ENT specialists at Dr. Sardjito Hospital, Yogyakarta Indonesia.

*RNA extraction and library preparation*

Total RNA from NPC samples and normal samples were isolated using the Rneasy® RNA Extraction Mini Kit (Qiagen, CA). The procedure for extraction follows the instructions in the kit. The cDNA library was developed using the TruSeq® RNA Library Preparation Kit V2 (Illumina, CA). The quantity and quality of the library are then tested using qPCR (Applied Biosystem, USA) and electrophoresis gel agarose.

*RNA-Seq*

The library sequencing was performed using Next Generation Sequencing (NGS) Illumina NexSeq 550 (CA) platform in Eijkman Institute, Jakarta. The quality of sequencing data by NGS machine was then tested using FastQC version 0.11.8 (Babraham Bioinformatics, UK). 

*RNA-Seq data analysis*

The raw sequencing data from the NGS machine were then interpreted using some bioinformatics software, including HISAT2 (Johns Hopkins Unversity, USA), HTSeq (EMBL Heidelberg, DEU), edgeR (National Health and Medical Research Council, AUS), and PANTHER (University of Southern California, CA). HISAT 2 (version 2.1.0) is used for aligning and mapping using the H. sapiens UCSC hg38 genome reference (https://ccb.jhu.edu/software/hisat2/index.shtml), HTSeq (version 0.11.1) is used to count the gene transcripts based on H. sapiens Ensembl database (ftp://ftp.ensembl.org/pub/release-98/gtf/homo_sapiens). edgeR (R/Bioconductor package version 3.28.0) is used for differential gene expression analysis, and PANTHER (version 14.1) is used for pathway analysis.

## Results

*Library preparation*

**Figure 1:**
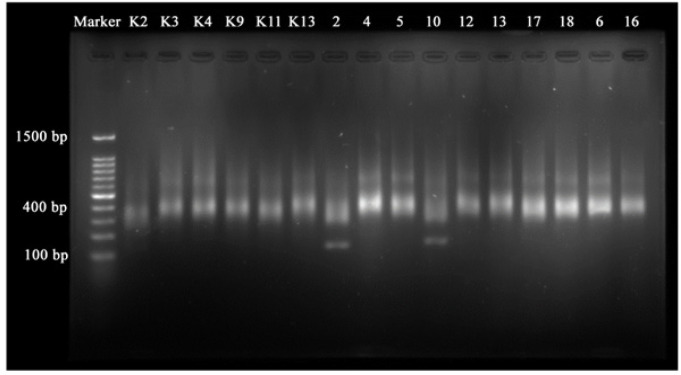
Electrogram of cDNA Library Fragment

The library preparation is divided into; ribosomal RNA (rRNA) removal and RNA fragmentation, cDNA synthesis, and library validation. rRNA must be removed because it is the type of RNA that is most present in cells but is not needed for transcriptomic analysis, while RNA fragmentation has to be completed because the NGS machine has limitations in reading long sequences. The short RNA fragment allows the NGS machine to read sequences correctly (Hrdlickova et al., 2017). Synthesis of cDNA is needed to convert RNA to complement DNA (cDNA). After synthesis, the cDNA library is then validated using quantitative PCR (qPCR) to calculate the concentration of cDNA produced in the reverse transcription process. cDNA concentration can be seen in Table 1. After calculating the quantity of cDNA, cDNA fragments were measured using agarose gel electrophoresis techniques. The results show that all samples size are 400 bp, which can be seen in Figure 1. 

*RNA-Seq*

Sequencing method using the NGS Illumina NextSeq 550 platform with paired-end sequencing protocol, the sequencing process occurs at both ends of the sequence (read 1 and 2). The length of the reads (fragments) of each sample ranged from 35 to 76 bp, with the total length of the sequences and the total percentage of bases guanine and cytosine varying each sample. The raw data produced by the NGS machine is then processed using a quality control tool, FastQC to assessing read quality. The result based on software provides good quality on each sample, so no trimming process needed to cut a low-quality sequence, and the raw data could be used for further analysis. The details can be seen in Table 2.

*Differential gene expression analysis*

**Figure 2:**
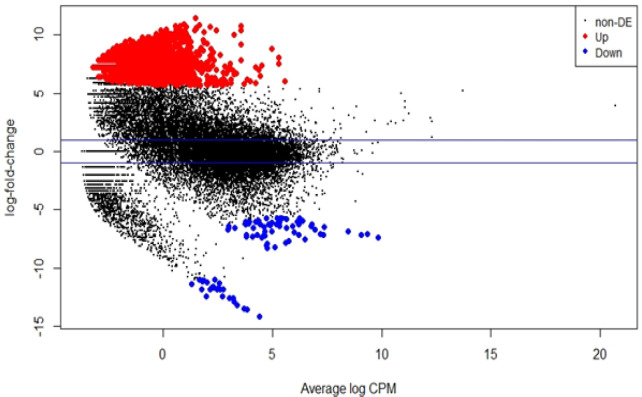
Differential Gene Expression Plot Data. The red dots represent the upregulated genes, the blue dots represent the downregulated genes, and the black dots represent the genes that didn’t significantly change the expression level (P<0.05).

The output from the NGS machine is raw data (data.fastq) that must be processed using some bioinformatics software. The HISAT2 software is used to aligning and mapping to genome reference. The extension data generated by HISAT2 is data.sam. Then HTSeq is used to count the gene transcript fragments per sample. The extension data generated by HTSeq is data.csv. The edgeR used to calculate the differential gene expression level. The results showed that there were 25,493 gene transcripts were expressed, with 1956 genes were upregulated, 90 genes were downregulated significantly in NPC samples compared to controls (Supplementary 1), and 23,897 gene transcripts didn’t change the expression level significantly (P<0.05). A list of genes that significantly upregulated or downregulated and its fold change can be seen in Table 3, while the visualization plot data can be seen in Figure 2. From several genes that changed the expression level significantly, 10 of these genes are related to cell proliferation, with details in Table 4.

**Table 1:** The Results of Measurements of cDNA Concentration and cDNA Fragments Size

Sample	cDNA concentration (pM)	cDNA fragment size (bp)
K2	9,18	400
K4	5,23	400
4	4,73	400
5	11,82	400
10	12,77	400
12	6,28	400
13	9,65	400
6	14,44	400
16	27,61	400

**Table 2:** The Sequence Data Produced by NGS

Sample	Read	Total sequence (bp)	Sequence length (bp)	%GC	Sequence with poor quality
K2	1	33097617	35-76	53	0
	2	33097617	36-76	55	0
K4	1	169898666	35-76	50	0
	2	169898666	35-76	51	0
4	1	186979159	35-76	57	0
	2	186979159	35-76	58	0
5	1	20413210	35-76	56	0
	2	20413210	35-76	57	0
10	1	18128296	35-76	49	0
	2	18128296	35-76	52	0
12	1	87652547	35-76	56	0
	2	87652547	35-76	58	0
13	1	67777774	35-76	53	0
	2	67777774	35-76	54	0
6	1	36803557	35-76	53	0
	2	36803557	35-76	53	0
16	1	64223909	35-76	48	0
	2	64223909	35-76	49	0

**Table 3:** List of the Top 30 Genes that Upregulated and Downregulated Significantly

Gene (symbol)	Fold change (LogFC)	LogCPM	P-value	Differential expression
*GABRG2*	114,795	1,458,475	0,0000032	Upregulated
*SALL3*	1,093,225	0,94557	0,0000063	Upregulated
*HOXA-AS3*	1,087,569	1,637,053	0,0000068	Upregulated
*LINC01877*	1,075,546	0,555914	0,0000078	Upregulated
*KCNQ2*	1,075,546	0,758206	0,0000078	Upregulated
*SOX11*	1,069,137	2,123,834	0,0000085	Upregulated
*NEFM*	1,069,137	103,832	0,0000085	Upregulated
*IGFN1*	1,076,803	3,540,036	0,0000091	Upregulated
*LINC01630*	106,243	1,512,201	0,0000092	Upregulated
*HAND2-AS1*	1,055,396	1,522,132	0,0000100	Upregulated
*HOXB8*	1,048,001	0,140411	0,0000110	Upregulated
*CLVS2*	1,048,001	0,592014	0,0000110	Upregulated
*SIM1*	1,040,206	3,540,933	0,0000121	Upregulated
*NOVA2*	1,040,206	1,440,537	0,0000121	Upregulated
*LINC01608*	1,040,206	2,418,177	0,0000121	Upregulated
*HSD17B13*	-141,658	4,386,143	0,0000956	Downregulated
*DAW1*	-13,581	3,840,144	0,0001918	Downregulated
*SLC5A8*	-135,206	3,706,032	0,0002061	Downregulated
*CFAP100*	-131,998	3,379,963	0,0003017	Downregulated
*ADH1C*	-827,494	4,757,425	0,0003975	Downregulated
*GSTA2*	-820,274	5,092,261	0,0004334	Downregulated
*CES1*	-786,644	5,605,258	0,0004395	Downregulated
*TTC29*	-128,559	3,236,973	0,0004533	Downregulated
*SLC44A4*	-769,539	5,751,363	0,0004600	Downregulated
*AGR2*	-751,219	6,487,753	0,0004780	Downregulated
*MUC5AC*	-734,873	9,835,014	0,0004789	Downregulated
*TEKT1*	-806,593	4,718,404	0,0005107	Downregulated
*ENPP4*	-793,517	4,739,182	0,0005974	Downregulated
*EFCAB10*	-12,6	3,195,775	0,0006132	Downregulated
*BPIFB1*	-714,311	9,066,221	0,0006227	Downregulated

**Table 4:** List of Genes Related to Proliferation Pathway

Gene (symbol)	Differential expression	Fold change	P-value
*GCK*	Upregulated	5.94	0.003478
*SPANXA2-OT1*	Upregulated	7.19	0.000882
*PRKCG*	Upregulated	5.88	0.003569
*IGF2*	Upregulated	8.24	0.000221
*INSRR*	Upregulated	6.45	0.002699
*DLK1*	Upregulated	9.04	0.00007
*RBPJL*	Upregulated	9.4	0.000043
*HELT*	Upregulated	7.82	0.000426
*NOS2*	Downregulated	-7.13	0.001067
*IGFBP1*	Upregulated	7.24	0.00122

## Discussion

Cancer cell proliferation is mediated by several signaling pathways, including the Insulin-like growth factor (IGF) signaling pathway, Notch signaling pathway, P13 kinase (P13K) signaling pathway, and disruption of the apoptotic signaling pathway. Based on the analysis of PANTHER, 10 genes were associated with these pathways. 

In the apoptosis signaling pathway, there are 3 genes involved, including GCK, SPANXA2-OT1, and PRKCG. Apoptosis is a programmed cell death that activated when the cell proliferation system dysregulated, similar to cancer. The apoptotic signaling pathway will be activated intracellularly by activating caspase. The apoptotic signaling pathway began when ligand was released. The ligand binds to the death receptor (DR) located on the cell surface, such as tumor necrosis factor receptor (TNF), which includes TNF-R1, CD 95 (Fas), and TNF-related apoptosis-induced ligand (TRAIL)-R1 and R2. Ligands that bind to these receptors will trigger caspase 8 (caspase initiator) to form trimers with a Fas-associated death domain (FADD) protein adapter. CD 95, TRAIL-R1 and R2 receptors will bind to FADD, while TNF will bind indirectly to TNF-associated death domain (TRADD) adapters,. The complex formed by the ligand-receptor binding is called DISC; this complex will activate pro-caspase-8 to become active caspase 8 (Kuntz et al., 1999). Caspase 8 then cut the Bcl-2 family member, Bid. The severed bid will induce Bax insertion into the mitochondrial membrane and release the cytochrome-C that are apoptotic molecules to the cytosol. Cytochrome-C will bind to Apaf-1 to form a caspase recruitment domain (CARD). Some CARDs form the apoptosome complex then bind to pro-caspase-9 and activate it into caspase 9 (caspase initiator). Caspase-9 will activate pro-caspase-3 to caspase-3 which is an effector of apoptosis (Wong, 2011). Caspase 3 can also activate other caspases, such as caspase 6 and 7 which provide an amplification process for cellular damage. The presence of cellular damage will increase protein expression, which results in G1 arrest or apoptosis (Rastogi et al., 2009).

GCK is a gene that codes germinal center kinase protein (GCK). GCK is involved in several cellular processes, including triggering cell growth and proliferation, cell polarity, cell migration, and is involved in response to stress. In general, GCK plays a role in activating the ERK, JNK, and p38 MAPK kinase signaling pathways (Schouest et al., 2009). Several types of GCK from different species can induce apoptotic programs. According to Matthews et al., (2013), GCK activation contributes to triggering cell proliferation and resistance, so inhibition of GCK can inhibit cell growth and proliferation. Based on the analysis, CGK in NPC samples are upregulated, indicates that in NPC cases, cancer cells have grown and proliferated and there is no inhibition mechanism in the apoptotic signaling pathway. 

SPANXA2-OT1 has been upregulated in this study. According to the human gene database (GeneCards), SPANXA2-OT1 is a non-coding protein, whose function is not yet known. Meanwhile, according to PANTHER, SPANXA2-OT1 plays a role in the signaling pathway of the BCL-2 protein group, which functions as an anti-apoptotic protein. Increased expression of SPANXA2-OT1 in NPC samples indicates that NPC cells experience inhibition of the apoptosis process, resulting in increased cancer cell proliferation.

PRKCG is a gene that encodes gamma C protein kinase (PKC). PKC acts as a tumor promoter. An increase in the expression level of this protein will enhance cell adhesion and proliferation, and inhibit the apoptosis process while a decrease in the expression level of this protein will reduce cell migration and formation (Downling et al., 2017). The upregulated expression of PRKCG in NPC samples indicates that cell proliferation was increased and the apoptotic process was inhibited in NPC cases.

In the IGF signaling pathway, there are 2 genes involved in this pathway, including IGF2 and INSRR. IGF signaling pathways play an essential role in regulating the growth, development, and maintenance of the cells in human body tissues. This signaling pathway plays a role in stimulating cell proliferation and inhibits apoptosis. This signaling system depends on 2 ligands, such as IGF-1 and IGF-2, which will bind to IGF-1R receptors (primary), IGF-2R, and insulin receptors, which are all included in the tyrosine kinase group (1,2). After the ligand binds to the receptor, IGF-1R will be activated via autophosphorylation and will phosphorylate the insulin receptor substrate 1 (IRS-1). So the phosphoinositide 3-kinase (P13K) remains active and increases the phosphatidylinositol 3,4,5-triphosphate (PIP3). Then the Akt/PKB protein is activated through phosphorylation. Akt plays a role in several functions, including releasing the Bcl-2 protein which is an anti-apoptotic protein from Bad, activating protein synthesis through mTOR, and increasing glucose metabolism by inhibiting the glycogen synthase kinase 3 beta (GSK-3β) (Zha and Lackner, 2010; Siddle, 2011), which is included in the P13K/Akt pathway of the IGF-1R signaling pathway that responsible for inhibiting the apoptotic process (Kennedy et al., 1997). In parallel, IGF-1R signaling also triggers cell proliferation via the Ras/MAPK signaling pathway (Zha and Lackner, 2010). IGF-1R activates the IRS SHC protein, which then stimulates Raf through Ras GTPase. Raf will then trigger a cascade of kinases, which will ultimately result in the activation of mitogen-activated protein kinases (MAPKs), ERK1, and ERK2. This MAPK will continue phosphorylated and activated via several target proteins, especially the ELK1 transcription factor that plays a role in increasing gene expression, thereby increasing cell growth and proliferation (Pouyssegur et al., 2002; Taniguchi et al., 2006).

IGF2 encode insulin-like growth factor-2 (IGF-2). This protein plays an important role in inducing cell proliferation in many different tissues. IGF-2 is the ligand of IGF-1R and INSR receptors, that will activate the proliferation process through the intracellular phosphorylation cascade, which belongs to the ERK/MAPK pathway and other pathways that regulate the progression of cell division and proliferation (Li et al., 2011). The upregulate of IGF2 expression in NPC samples showing that cell proliferation is increased on NPC cases.

INSRR encodes the protein related to the insulin receptor. Insulin receptors will bind to insulin ligands, like IGF, which will eventually induce cell proliferation (Li et al., 2011). When insulin binds to the insulin receptor, protein kinase will be active and produce phosphorylation of tyrosine residues in the β-subunit, which will lead to the recruitment of several adapter proteins, including the src-homology 2 (SH2) domain, which contains proteins such as P13K and phospholipase C (PLC). PLC will hydrolyze phosphatidylinositol 4,5-bisphosphate (PIP2), which will produce the formation of diacylglycerol (DAG), so the PKC and inositol-1,4,5-trisphosphate (InsP3) will active and trigger the release of Ca2+ ions when binding to the InsP3 receptor (InsP3R). The release of Ca^2+^ is essential for the process of cell proliferation, especially during the early prophase (Amaya et al., 2014). An upregulated expression of the INSRR showed that there is increased cell proliferation in the case of NPC.

In the Notch signaling pathway, there are 3 genes involved, they are *DLK1*, *RBPJL*, and *HELT*. The notch signaling pathway plays a role in regulating cell proliferation, differentiation, and resistance. This signaling pathway is commonly involved in the incidence of cancer. The two groups of Notch ligands are Jagged-1 and 2 (JAG1 and JAG2) and also Delta-like 1,3, and 4 (DLL 1, DLL3, and DLL 4). Notch receptors consist of Notch 1,2,3, and 4 (Huang et al., 2019). When the ligand binds to the receptor, the complex will induce the unfolding of the juxtamembrane negative control region (NRR) to the Notch protein. The opening of the NRR fold causes ADAM 10 (including the protease group) to access the NRR, which can eliminate the extracellular domain of Notch by cutting it at site 2 (S2): secret-secretase, then cut Notch in its transmembrane domain at site 3 (S3) to release Notch intracellular domain (NICD). NICD will be released into the nucleus and will interact with DNA-binding CSL proteins. This mechanism will activate the transcription system of the Notch target gene, including members of the hairy and enhancer of split 1 (HES1) and HES1-related (HESR1) transcription factors (Nowell and Ratdke, 2013). These transcription factors control some cellular processes, including proliferation, differentiation, and deregulation of apoptosis in cancer development and progression (Sonoshita et al., 2015).

DLK1 encodes delta like non-canonical Notch ligand 1 protein, a transmembrane protein that contains multiple epidermal growth factors which function is to regulate cell growth for cell differentiation. This protein acts as an inhibitor of the Notch signaling (in vitro). DLK 1 inhibits the attachment of JAGs (ligands) to the Notch receptor (Falix et al., 2012). However, the in vivo function isn’t yet clearly understood because DLK1 is also expressed in many embryonic tissues when the Notch signaling pathway is also active (Falix et al., 2012). An upregulated expression of DLK1 in NPC indicates that cells regulate to block the Notch signaling pathway so it can inhibit cell proliferation. The high-level expression of DLK1 is a survival response indicator of NPC patients.

RBPJL encodes the recombining binding protein suppressor of hairless-like protein (RBPJL). RBPJL is a transcription mediator of DNA binding protein in the Notch signaling pathway (Masui et al., 2010). RBPJL is a transcription factor which in the promoter area will bind to the NICD and will activate the transcription. So that the Notch signaling pathway will be activated (Nair et al., 2018). An upregulated expression of RBPJL indicates that the Notch signaling pathway is active in the case of NPC, and it will increase the proliferation and progression of cancer cells. 

HELT (Hairy and enhancer of split-related protein HELT) is a gene that codes for the Helt BHLH transcription factor. This protein can form a heterodimer and binds the Ebox in the promoter. The Ebox sequences are needed for the introduction of HELT and HES1 proteins. Furthermore, HELT will suppress the transcription of other promoters through optimal binding to the Ebox elements (Nakatani et al., 2004). Based on PANTHER, the transcription of HELT is regulated by the output product from the Notch signaling pathway. So the upregulated expression of HELT in NPC samples indicating that the Notch signaling pathway was active under the NPC conditions.

In the P13K signaling pathway, there are 2 genes involved, including *NOS2* and *IGFBP1*. The P13K/Akt signaling pathway is a metabolic pathway that induces cell proliferation, cell growth, survival, and angiogenesis. This signaling pathway begins when growth factor ligands attach to the tyrosine kinase (RTK) receptor. Two adjacent RTK monomers then form dimers, which will trigger the activation of the tyrosine kinase protein group and will be autophosphorylated. Furthermore, the P13K signaling pathway will be active through the insulin receptor substrate (IRS) or the Ras-GTP complex. Then PIP3 which is a minor phospholipid in the cell membrane will bind to a specific domain, which triggers the release of protein to the plasma membrane that can activate a cascade signal. There are 3 phosphate group positions in PIP3 that bind to phosphoinositide-dependent protein kinase 1 (PDK1) and Akt protein so that Akt 1 protein will be released to the plasma membrane and PDK1 can be accessed. Then T308 phosphorylation occurs in the activation loop, which causes the activation of PKB/Akt. Akt will be more active when phosphorylation occurs at S473. Activation of the Akt will activate several cellular processes such as cell growth and proliferation (Hemmings and Restuccia, 2015).

NOS2 encodes nitric oxide synthase 2 protein (NOS2). According to GeneCards, NOS2 is a reactive free radical that acts as a mediator in some biological processes, such as a neurotransmitter, an anti-microbial, and an anti-tumor. This protein mediates bactericidal and tumoricidal processes. NOS2 is an antitumor component as part of the immune response, which has a low expression in cancer (Thomas and Wink, 2017). In this study, the expression of NOS2 is downregulated in NPC samples. It’s indicated that in the NPC cases, there is a decrease in antitumor activity that will enhance the growth and progression of the tumor.

IGFBP1 encodes insulin-like growth factor binding protein 1 (IGFBP1). IGFBP1 plays an essential role in the development and progression of cancer (Dai et al., 2014). IGFBP1 is one of 6 binding protein that is soluble and can affect the bioactivity of IGF. IGFBP can bind IGF with high binding affinity, thus blocking the binding of IGF to its receptor (IGF-R). However, IGFBP can also increase the IGF activity depending on the regulation of IGFBP protease. IGF can also act as a strong mitogen and as an anti-apoptosis in various types of cancer. But IGF can also play a synergy role with mitogen and steroid growth factors as an antagonist to anti-proliferation molecules in cancer growth (Yu and Rohan, 2000). The upregulated expression of IGFBP1 in NPC samples indicates that in the NPC cases, there are some regulations that cause the increased proliferation of cancer cells.
